# Supplementary figures and images for: Unified extractive-abstractive summarization: a hybrid approach utilizing BERT and transformer models for enhanced document summarization
Source: PeerJ Comput Sci. 2024 Nov 18;10:e2424. doi: 10.7717/peerj-cs.2424 (PMC11802190; doi:10.7717/peerj-cs.2424)

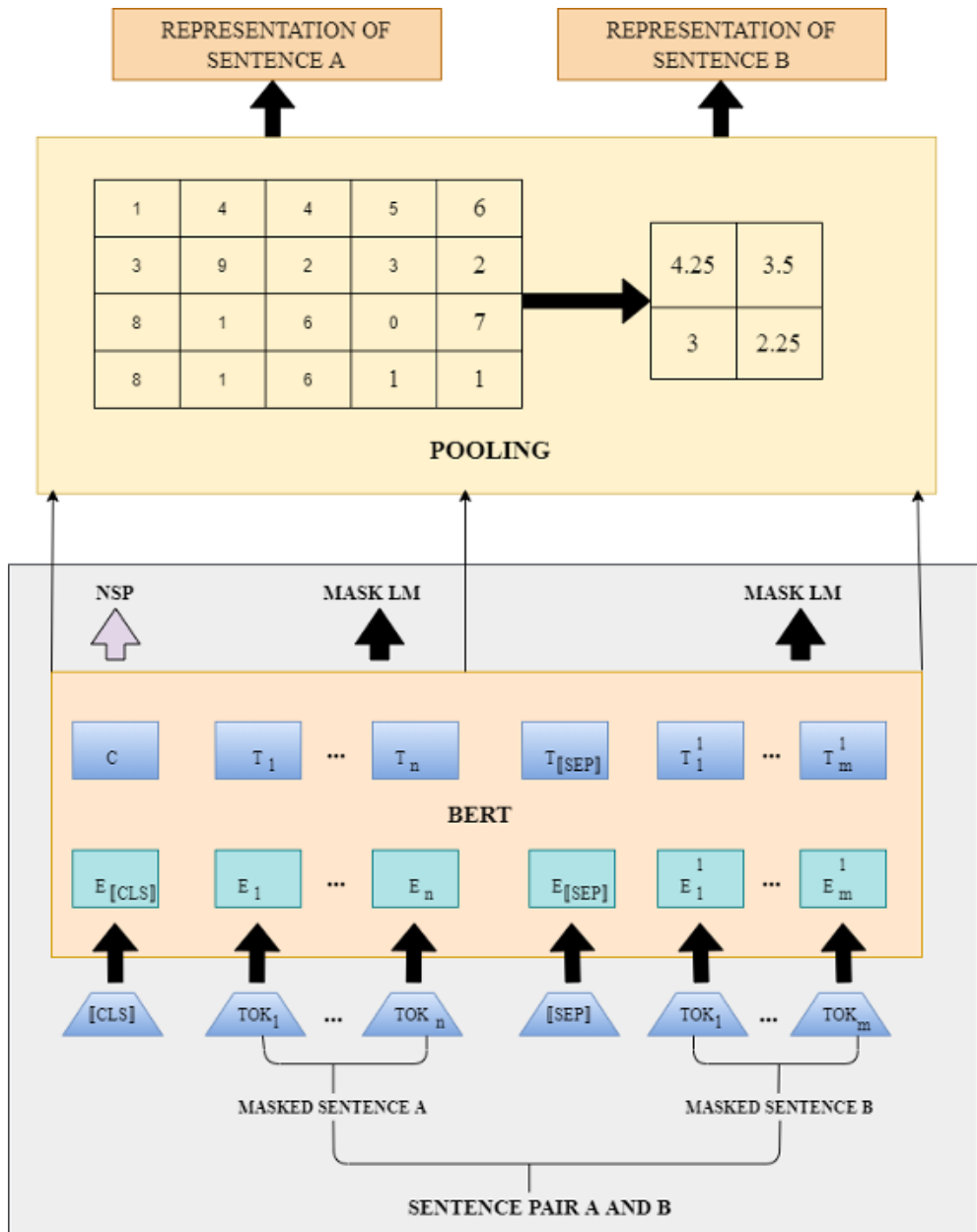

Supplement: Supplemental Information 1 — This explains the steps involved in the extraction of informative sentences for the generation of extractive summary. [file peerj-cs-10-2424-s001.pdf]

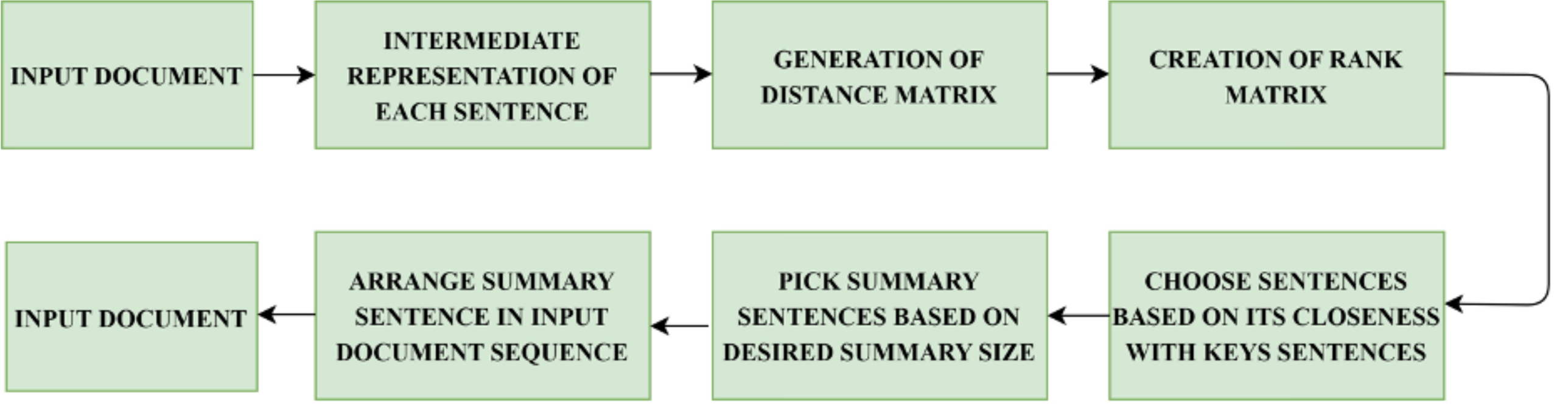

Supplement: Supplemental Information 2 — The functional flow which represents the generation of intermediate representation for each sentence using BERT is detailed. [CLS] label is appended in the beginning of each sentence and two sentences are separated using [SEP] token. Each token in the sentence is considered as input to the embedding layer. These n dimensional vectors are given as input for the pooling layer to generate representation for each sentence. [file peerj-cs-10-2424-s002.png]
