# Supplementary material for: Unified extractive-abstractive summarization: a hybrid approach utilizing BERT and transformer models for enhanced document summarization
Source: PeerJ Comput Sci. 2024 Nov 18;10:e2424. doi: 10.7717/peerj-cs.2424 (PMC11802190; doi:10.7717/peerj-cs.2424)
Supplement: Supplemental Information 4 [file peerj-cs-10-2424-s004.docx]

1. create virtual environment

Python -m venv venv

2. installing requirements from file

pip install -m requirements.txt

3. run python file
 python abstractive_summarization.py
